# Characterization of rotavirus infection in children with acute gastroenteritis in Bengo province, Northwestern Angola, prior to vaccine introduction

**DOI:** 10.1371/journal.pone.0176046

**Published:** 2017-04-19

**Authors:** Carolina Gasparinho, João Piedade, Maria Clara Mirante, Cristina Mendes, Carlos Mayer, Susana Vaz Nery, Miguel Brito, Claudia Istrate

**Affiliations:** 1 Centro de Investigação em Saúde de Angola (CISA), Caxito, Província do Bengo, Angola; 2 Global Health and Tropical Medicine (GHTM), Unidade de Microbiologia Médica, Instituto de Higiene e Medicina Tropical (IHMT), Universidade NOVA de Lisboa (UNL), Lisbon, Portugal; 3 Hospital Geral do Bengo, Caxito, Província do Bengo, Angola; 4 Research School of Population Health, The Australian National University, Canberra, Australia; 5 Escola Superior de Tecnologia da Saúde de Lisboa, Lisbon, Portugal; Universidad Nacional de la Plata, ARGENTINA

## Abstract

**Background:**

Rotavirus group A (RVA) is considered the leading cause of pediatric diarrhea, responsible for the high burden of diarrheal diseases in sub-Saharan Africa. Despite recent studies, the existent data are scarce for some African countries like Angola, a country with one of the highest RVA-related death estimates. The aim of this study was to determine the RVA detection rate and circulating genotypes in children less than five years of age with acute gastroenteritis attended at the Bengo General Hospital in Caxito, Bengo province, Angola, before vaccine introduction.

**Methods:**

Between September 2012 and December 2013, 342 fecal specimens were collected from children enrolled. Positive samples for RVA by immunochromatographic rapid test were G and P-typed by hemi-nested type-specific multiplex PCR, and subgrouped for the VP6 gene. VP4 and VP7 genes from a subset of samples were sequenced for phylogenetic analysis.

**Results:**

During the study period, a high RVA detection rate was registered (25.1%, 86/342).

The age group most affected by RVA infection includes children under 6 months of age (p<0.01). Vomiting was highly associated with RVA infection (72.1%; p<0.001).

From the 86 RVA-positive samples, 72 (83.7%) were genotyped. The most prevalent genotype was G1P[8] (34/72; 47.2%), followed by the uncommon G1P[6] (21/72; 29.2%), and G2P[4] (9/72; 12.5%). Only two G-types were found: G1 (60/72; 83.3%) and G2 (11/72; 15.3%). Among the P-genotypes, P[8] was the most prevalent (34/72; 47.2%), followed by P[6] (22/72; 30.6%) and P[4] (9/72; 12.5%). In the phylogenetic trees, the identified G and P-types clustered tightly together and with reference sequences in specific monophyletic groups, with highly significant bootstrap values (≥92%).

**Conclusion:**

This pre-vaccination study revealed, for the first time for Bengo province (Angola), the RVA genotype profile, including phylogenetic relationships, and a high RVA detection rate, supporting the immediate introduction of a RVA vaccine in the national immunization programme.

## Introduction

Rotavirus group A (RVA) remains the most important etiological agent of severe diarrhea in children under five years of age [1, 2], especially in remote areas with difficult or inexistent access to the healthcare infrastructure and inadequate domestic sanitation conditions [2, 3].

According to the global estimates from 2008, RVA was responsible for 453,000 deaths among children under five years of age each year, with African children accounting for more than 50% of the total [4], but a recent study showed a decline in this number, up to 215,000 deaths in 2013, the majority of them in India [5]. In the last decade, an increased research effort on epidemiology and disease burden of RVA infection has been made. Data obtained helped in the definition of more effective health policies, including the implementation of RVA vaccination. Two live attenuated RVA vaccines, the monovalent Rotarix (GlaxoSmithKline Biologicals, Belgium) and the pentavalent human-bovine reassortant RotaTeq (Merck, USA), were recommended by the World Health Organization (WHO) to be included in the national immunization programmes worldwide in 2009 [6]. Recently, a third RVA vaccine, the low-cost, live attenuated Rotavac (Bharat Biotech International, India) was licensed for use in India but not yet pre-qualified for the Global Alliance for Vaccines and Immunization (GAVI) market [7]. Both recommended vaccines require multiple dose administration (two doses for Rotarix and three for RotaTeq), the first to be administered between 6 and 15 weeks of age [8], and raise homo- and heterotypic immune response against RVA different strains [9]. The two vaccines have been proven to be effective worldwide, but lower efficacy was observed in low-income countries from Africa and Southern Asia [10, 11]. Among the several hypothesis to explain the differences in the immune response and consequent efficacy of these vaccines in low- *versus* high-income countries, RVA strains diversity, host genetic factors, malnutrition, host co-infection, deficient micronutrient ingestion, and interfering gut flora have been put forward [12–14].

Worldwide, there is a great genetic diversity of circulating RVA wild-type strains. Several studies conducted in Africa identified G1P[8] as the most frequent RVA strain, while the most common strains for high- and middle-income countries in the *pre* vaccination era, G2P[4], G3P[8], G4P[8], and G9P[8] [15], have also been identified, but to a much lesser extent [16, 17]. Noteworthy, is the emergence of uncommon strains, such as G1P[6], G8P[6], G6P[6], G8P[8], G12P[6], and mixed G and P-strains, in sub-Saharan Africa [18–21]. This picture is still far from completed for the majority of African countries, e.g. Angola. Despite the recent publication of an RVA epidemiology study in four provinces of Angola [22], present data on RVA prevalence and genotype distribution remain scarce.

Angola is a sub-Saharan African country with an estimated population of 25.8 million in 2014. The Angolan population is very young, with an average age of 20.6 years and a proportion of 47.3% aged 0–14 years [23]. Angola has one of the highest RVA attributable death rate in children under five years (5% of global total) [5]. Bengo province, situated in Northwestern Angola and close to the capital city, Luanda, has a population of about 357.000 individuals, with 56.3% of them living in rural settings [24]. The capital, Caxito, harbor the Bengo General Hospital (BGH), a reference hospital for the province. To reduce severe diarrheal disease, and preventing RVA-associated hospitalizations and mortality, Angola requested assistance from the GAVI in 2014 to introduce RVA vaccine in the national immunization program.

This study is part of a larger investigation regarding diarrhea burden in children under five years in the Bengo province, Angola [25], and aims to provide baseline information on RVA infection before vaccine introduction. This hospital-based study was established to investigate RVA prevalence and genotype distribution during a period of more than one year in children up to five years attended with acute gastroenteritis (AGE).

## Material and methods

### Setting or study site

This study was conducted at the Bengo General Hospital (BGH), located in Caxito, capital of Bengo province, 60 km northeast of the capital Luanda. Besides receiving patients from Bengo province, the BGH also attends patients from neighboring Luanda province. The climate of this province is subtropical, characterized by a rainy and warm season, from mid-September to mid-May, and a dry and cold season, from mid-May to mid-September.

### Study design

This study was conducted at the BGH between September 2012 and December 2013 and is part of a cross-sectional study set up to investigate the most frequent etiological agents of diarrhea (viruses, parasites and bacteria) in children under five years of age [25]. Surveillance data was collected before rotavirus group A (RVA) vaccine introduction in the country. A total of 342 children with acute gastroenteritis AGE (diarrhea, with or without vomiting) attending the pediatric emergency room or the outpatient pediatric unit were enrolled and tested for RVA infection. During the sampling period, all children with AGE presenting at BGH, originating from Bengo province (n = 286) or Luanda province (n = 56), were included in this study. Diarrhea was defined as three or more loose or liquid stools per day [26]. Children receiving antibiotic or antiparasitic treatment within 10 days before the examination were excluded from the study. After informed consent was given by parents or legal guardians, sociodemographic variables [i.e. date of birth (n = 342), gender (n = 342), province of residence (n = 342), residence type (n = 250), maternal literacy (n = 337), education level of the mother (n = 272)], and information on breastfeeding practices (n = 240), drinking water source (n = 342), water treatment methods (n = 342) and sanitation conditions (n = 342) were obtained, whenever possible. Nutritional status was also assessed from anthropometric measurements (n = 332). Weight and length/height were measured according to standard procedures established by the World Health Organization (WHO) and used to calculate anthropometric indices expressed as individual *z* scores using ANTHRO software (version 3.2.2) [27]: weight-for-age (WAZ, underweight), weight-for-height (WHZ, wasting) and height-for-age (HAZ, stunting). Malnutrition was classified as mild (-2 ≤ *z* score < -1), moderate (-3 ≤ *z* score < -2) or severe (*z* score < -3) [28]. Children with symptoms of bilateral pitting edema were diagnosed as suffering from severe acute malnourishment as described in WHO procedures [29].

After physical examination, the clinical variables regarding symptoms associated to AGE were registered [e.g. duration of diarrhea in days (n = 333), vomiting (n = 342), fever (n = 342), lethargy (n = 325), dehydration signs, such as depressed fontanelle (n = 320), sunken eyes (n = 329), skin elasticity (n = 318)]. Depending upon the severity of symptoms, each child was referred for regular follow-up treatment (oral rehydration, drugs, etc.) or hospitalization. The type of admission was registered [e.g. outpatient department (n = 164) or emergency unit (n = 178)].

### Ethical considerations

The study protocol (including detailed working plan, epidemiological survey and informed consent forms) was approved by the National Ethics Committee of the Angolan Ministry of Health in Luanda and the Ethics Committee of the Institute of Hygiene and Tropical Medicine, in Lisbon, Portugal (Process number:12-2012-PN). As stated before, informed and voluntary written consent was obtained from parents or legal guardians of each child prior to inclusion in the study.

### Sample collection and RVA antigen detection

Fecal specimens were collected in sterile containers provided by clinical staff. A total of 342 samples were screened locally for the presence of RVA and adenovirus serotype 40/41 antigens using a rapid qualitative immunochromatographic assay (Rotavirus + Adenovirus, CerTest Biotec S.L., Zaragoza, Spain), following the manufacturer’s instructions. RVA-positive stool samples were preserved in guanidine thiocyanate solution until RNA extraction as described before [30].

### Viral RNA extraction

Preserved stool samples were transported to the Institute of Hygiene and Tropical Medicine in Lisbon, Portugal for RVA genotyping. Viral RNA was extracted from 10% (w/v) stool suspensions using the innuPREP Virus RNA Kit (Analytik Jena AG, Jena, Germany), according to the manufacturer’s instructions. The RNA was eluted in RNase-free water (60 μl) and stored at -80°C until further use.

### Reverse transcription, VP6 subgrouping, G and P-genotyping

Reverse transcription (RT) with random hexamers, to produce cDNA to be used as template in specific PCRs for the different genes, was carried out using a commercial kit (NZY First-strand cDNA Synthesis Kit, NZYTech, Lisbon, Portugal), according to the manufacturer’s instructions. Briefly, 16 μl of the RNA eluate was denatured at 94°C for 5 minutes, and quickly chilled on ice for 2 minutes, followed by the addition of 20 μl of NZYRT 2x Master Mix and 4 μl of NZYRT Enzyme Mix, to a final volume of 40 μl. The RT reaction was carried out at 25°C for 10 minutes, and 50°C for 30 minutes, being the inactivation made at 85°C for 5 minutes. After the final addition of 2 μl of NZY RNase H (*E*. *coli*), the product was incubated at 37°C for 20 minutes.

VP6 subgrouping was performed using conventional PCR, previously described in 2010 by Thongprachum et al. [31].

RVA G and P-genotyping were done using hemi-nested type specific multiplex PCRs, optimized to detect eight G-types (G1, G2, G3, G4, G8, G9, G10 and G11) and six P-types (P[4], P[6], P[8], P[9], P[10] and P[11]), as described previously [32–34]. The G- and P-genotypes were assigned according to the amplicon size visualized under ultraviolet light after electrophoresis on 2% agarose gels stained with ethidium bromide.

### Molecular characterization of RVA strains by phylogenetic analysis

First-round PCR amplicons for VP7 and VP4 genes from a set of randomly selected G- and P-typed viruses from each detected genotype, as well as from non-typable strains, were sent for DNA sequencing (Sanger method) for further molecular characterization. DNA sequencing was performed using the corresponding first-round PCR primers by STAB VIDA (Caparica, Portugal).

After sequence editing using the BioEdit Sequence Alignment Editor version 7.1.3.0 [35], multiple sequence alignments were made with Clustal Omega (available at http://www.ebi.ac.uk/Tools/msa/clustalo/). Phylogenetic analysis was performed with the MEGA 5.1 software [36], using a distance-based neighbour-joining method, based on the Kimura 2-parameter model [37]. Bootstrap values were calculated from 1,000 replicates [36].

### Nucleotide sequence accession numbers

The GenBank/DDBJ/EMBL accession numbers for sequences obtained in this study are KP216531-KP216547, for VP4, and KP216548-KP216561, for VP7.

### Statistical analysis

Data were analyzed using IBM SPSS software, version 22 (IBM Corp, Armonk, NY, USA). Absolute (n) and relative frequency (%) were used for descriptive statistics of categorical and ordinal variables, and the mean and standard deviation (SD) were presented in the case of continuous variables.

The chi-squared (χ2) test or Fisher’s exact test (for tables with expected cell frequencies less than 5) were used to compare categorical variable proportions (gender, group age, province of residence, settlement type, maternal literacy and education level, drinking water source and treatment method, sanitation facilities, breastfeeding, malnutrition and clinical symptoms) between RVA-positive and RVA-negative children. A p-value less than 0.05 was considered to be significant and associations were expressed in odds ratio (OR) and respective 95% confidence intervals (95% CI). A multiple logistic regression model was applied for stunting (dependent variable), and included the independent variables age, gender, infection with RVA and infection by an enteric pathogen other than RVA (data from the cross-sectional study published before [25]). The goodness of fit was based on Hosmer and Lemeshow test, considering a *p* value greater than 0.05. Student´s *t*-test was applied to compare the mean age (in months) between RVA-positive and RVA-negative children. When the p-value was less than 0.05, the mean age between the two groups were considered significantly different.

## Results

### RVA detection rate and basic socio-demographic characterization of the studied population

From September 2012 to December 2013, 342 children under five years of age with acute gastroenteritis (AGE), attended at the Bengo General Hospital (BGH), were tested for rotavirus group A (RVA) and enteric adenovirus (AdV) type 40/41 infections. RVA detection rate was 25.1% (86/342), while for AdV type 40/41 was much lower, reaching 3.8% (13/342). Two children were shown to have mixed infection (0.6%, 2/342).

No association was found between infection by RVA and children gender (Table 1). The mean age was significantly lower for children with RVA infection as detected by the RVA antigen immunochromatographic assay (9.2±5.00 *versus* 17.6±13.40 months, p<0.001)—Table 1. The age distribution is significantly different between RVA infected and non-infected children (p<0.001, Table 1), with a positive association between infection and younger ages (<12 months).

**Table 1:** Characteristics of the study sample.

		RV (+)	RV (-)	*p-value*	Total
		n (%)	n (%)		n (%)
**Study sample**		86 (25.1)	256 (74.9)		342 (100)
**Gender**	Male	49 (57.0)	134 (52.3)	0.456	183 (53.5)
	Female	37 (43.0)	122 (47.7)		159 (46.5)
**Mean age** (months)		9.2±5.00	17.6±13.4	**<0.001**	15.5±12.39
**Group age** (months)	[0–6[	21 (24.4)	34 (13.3)	**<0.001**	55 (16.1)
	[6–12[	48 (55.8)	84 (32.8)		132 (38.6)
	[12–24[	16 (18.6)	76 (29.7)		92 (26.9)
	[24–59]	1 (1.2)	62 (24.2)		63 (18.4)

Although BGH is a reference hospital for the province, 16.4% (56/342) of the attended children were residents of the neighbor Luanda province (Table 2). The vast majority of the enrolled children were living in an urban area (92.4%, 231/250), with only 7.6% (19/250) living in rural settlements (Table 2). When asked about maternal education, the majority of the respondents (65.0%, 219/337) declared as graduating basic education, only 14.2% (48/337) frequented the high school, and 1.5% (5/337) had a university degree. 19.3% (65/337) of the mothers never attended school. No significant association was found between level of maternal literacy or education and RVA infection or the age of children enrolled. Apart from age and age distribution, there was no statistically significant association between RVA infection and any other socio-demographic variable analyzed.

**Table 2:** Sociodemographic profile of children with diarrhea attended at the Bengo General Hospital.

Variable	[0–12 [months		[12–24 [months		[24–59] months		[0–59] months	
	RV(+)	RV(-)	RV(+)	RV(-)	RV(+)	RV(-)	RV(+)	RV(-)
	n (%)	n (%)	n (%)	n (%)	n (%)	n (%)	n (%)	n (%)
**Child gender** (N = 342)	N = 69	N = 118	N = 16	N = 76	N = 1	N = 62	N = 86	N = 256
Male (N = 183)	39 (56.5)	72 (61.0)	9 (56.3)	34 (44.7)	1 (100.0)	28 (45.2)	49 (57.0)	134 (52.3)
Female (N = 159)	30 (43.5)	46 (39.0)	7 (43.8)	42 (55.3)	0 (0.0)	34 (54.8)	37 (43.0)	122 (47.7)
**Province of residence** (N = 342)	N = 69	N = 118	N = 16	N = 76	N = 1	N = 62	N = 86	N = 256
Bengo (N = 286)	54 (78.3)	100 (84.7)	14 (87.5)	64 (84.2)	1 (100)	53 (85.5)	69 (80.2)	217 (84.8)
Luanda (N = 56)	15 (21.7)	18 (15.3)	2 (12.5)	12 (15.8)	0 (0.0)	9 (14.5)	17 (19.8)	39 (15.2)
**Settlement type**^**a**^ (N = 250)	N = 49	N = 90	N = 8	N = 57	N = 1	N = 45	N = 58	N = 192
Urban (N = 231)	44 (89.8)	81 (90.0)	8 (100.0)	54 (94.7)	1 (100.0)	43 (95.6)	53 (91.4)	178 (92.7)
Rural (N = 19)	5 (10.2)	9 (10.0)	0 (0.0)	3 (5.3)	0 (0.0)	2 (4.4)	5 (8.6)	14 (7.3)
**Maternal literacy** (N = 337)	N = 69	N = 115	N = 16	N = 74	N = 1	N = 62	N = 86	N = 251
No (N = 65)	14 (20.3)	20 (17.4)	2 (12.5)	13 (17.6)	0 (0.0)	16 (25.8)	16 (18.6)	49 (19.5)
Yes (N = 272)	55 (79.7)	95 (82.6)	14 (87.5)	61 (82.4)	1 (100.0)	46 (74.2)	70 (81.4)	202 (80.5)
**Education level of the mother** (N = 272)	N = 55	N = 95	N = 14	N = 61	N = 1	N = 46	N = 70	N = 202
Basic (N = 219)	46 (83.6)	78 (82.1)	11 (78.6)	48 (78.7)	1 (100.0)	35 (76.1)	58 (82.9)	161 (79.7)
High school (N = 48)	6 (10.9)	16 (16.8)	3 (21.4)	13 (21.3)	0 (0.0)	10 (21.7)	9 (12.9)	39 (19.3)
University (N = 5)	3 (5.5)	1 (1.1)	0 (0.0)	0 (0.0)	0 (0.0)	1 (2.2)	3 (4.3)	2 (1.0)
**Water source from the CWTP**^**b**^ (N = 342)	N = 69	N = 118	N = 16	N = 76	N = 1	N = 62	N = 86	N = 256
Yes (N = 238)	51 (73.9)	80 (67.8)	12 (75.0)	50 (65.8)	1 (100.0)	44 (71.0)	64 (74.4)	174 (68.0)
No (N = 104)	18 (26.1)	38 (32.2)	4 (25.0)	26 (34.2)	0 (0.0)	18 (29.0)	22 (25.6)	82 (32.0)
**Drinking water source/treatment**^**c**^ (N = 342)	N = 69	N = 118	N = 16	N = 76	N = 1	N = 62	N = 86	N = 256
From CWTP and not treated at home (N = 98)	20 (29.0)	32 (27.1)	6 (37.5)	21 (27.6)	1 (100.0)	18 (29.0)	27 (31.4)	71 (27.7)
From CWTP and treated at home (N = 140)	31 (44.9)	48 (40.7)	6 (37.5)	29 (38.2)	0 (0.0)	26 (41.9)	37 (43.0)	103 (40.2)
Only treated at home (N = 65)	11 (15.9)	25 (21.2)	3 (18.8)	16 (21.1)	0 (0.0)	10 (16.1)	14 (16.3)	51 (19.9)
Not treated (N = 39)	7 (10.1)	13 (11.0)	1 (6.3)	10 (13.2)	0 (0.0)	8 (12.9)	8 (9.3)	31 (12.1)
**Sanitation facilities** (N = 342)	N = 69	N = 118	N = 16	N = 76	N = 1	N = 62	N = 86	N = 256
Without latrine (N = 70)	12 (17.4)	24 (20.3)	2 (12.5)	14 (18.4)	0 (0.0)	18 (29.0)	14 (16.3)	56 (21.9)
Private latrine with running water (N = 99)	18 (26.1)	39 (33.1)	4 (25.0)	20 (26.3)	1 (100.0)	17 (27.4)	23 (26.7)	76 (29.7)
Private latrine without running water (N = 47)	11 (15.9)	12 (10.2)	3 (18.8)	14 (18.4)	0 (0.0)	7 (11.3)	14 (16.3)	33 (12.9)
Public latrine with running water (N = 59)	15 (21.7)	18 (15.3)	6 (37.5)	11 (14.5)	0 (0.0)	9 (14.5)	21 (24.4)	38 (14.8)
Public latrine without running water (N = 67)	13 (18.8)	25 (21.2)	1 (6.3)	17 (22.4)	0 (0.0)	11 (17.7)	14 (16.3)	53 (20.7)
**Breastfeeding** (N = 342)	N = 69	N = 118	N = 16	N = 76	N = 1	N = 62	N = 86	N = 256
Exclusive (N = 38)	14 (20.3)	20 (16.9)	0 (0.0)	3 (3.9)	0 (0.0)	1 (1.6)	14 (16.3)	24 (9.4)
Complementary (N = 202	54 (78.3)	85 (72.0)	14 (87.5)	45 (59.2)	0 (0.0)	4 (6.5)	68 (79.1)	134 (52.3)
Weaned (N = 98)	1 (1.4)	11 (9.3)	2 (12.5)	28 (36.8)	1 (100.0)	55 (88.7)	4 (4.7)	94 (36.7)
Never (N = 4)	0 (0.0)	2 (1.7)	0 (0.0)	0 (0.0)	0 (0.0)	2 (3.2)	0 (0.0)	4 (1.6)
**Malnutrition** (N = 332)								
**- Underweight *(weight for age z score)***								
WAZ ≤ -2SD (N = 113)	23 (33.8)	36 (31.3)	9 (56.3)	25 (35.2)	1 (100.0)	19 (31.1)	33 (38.8)	80 (32.4)
WAS > -2SD (N = 219)	45 (66.2)	79 (68.7)	7 (43.8)	46 (64.8)	0 (0.0)	42 (68.9)	52 (61.2)	167 (67.6)
**- Wasting *(weight for height z score)***								
WHZ ≤ -2SD (N = 103)	26 (37.7)	32 (28.3)	5 (33.3)	26 (35.1)	1 (100.0)	13 (21.7)	32 (37.6)	71 (28.7)
WHZ > -2SD (N = 229)	43 (62.3)	81 (71.7)	10 (66.7)	48 (64.9)	0 (0.0)	47 (78.3)	53 (62.4)	176 (71.3)
**- Stunting *(height for age z score)***								
HAZ ≤ -2SD (N = 107)	10 (14.5)	37 (32.7)*	4 (26.7)	33 (44.6)	0 (0.0)	23 (38.3)	14 (16.5)	93 (37.7)**
HAZ > -2SD (N = 225)	59 (85.5)	76 (67.3)	11 (73.3)	41 (55.4)	1 (100.0)	37 (61.7)	71 (83.5)	154 (62.3)

^**a**^From the 342 households, only those from Centro de Investigação em Saúde de Angola (CISA) study area were included for analysis (N = 250).

^**b**^ Water from the Central Water Treatment Plant (CWTP) included drinking water from taps in the yard, private tanks, public taps and piped water. Other water sources, as the river, irrigation channel and borehole/tubewell were considered not being from the CWTP.

^**c**^ Water treatment methods at home included the use of bleach or alum stone, boiling and filtration.

* *p* value (*x*^*2*^) = 0.006; OR = 0.348; 95%CI] 0.160–0.757[

** *p* value (*x*^*2*^) <0.001; OR = 0.327; 95%CI] 0.174;0.612[

### Drinking water source and treatment method

The majority of the households with children participating in the study (69.6%, 238/342) used water supplied by the Central Water Treatment Plant (CWTP) (Table 2). However, in 58.8% (140/238) of these households an additional treatment, including the use of bleach and alum stone, boiling and/or filtration, was applied before drinking. The consumption of water only treated at home or without any type of treatment was reported, respectively, for 19.0% (65/342) and 11.4% (39/342) of the households.

Among all the RVA positive cases, 43.0% (37/86) of children were drinking water from the CWTP with a further household treatment, 31.4% (27/86) were using water from CWTP without any additional treatment. In 16.3% (14/86) of the cases, it was applied only a household treatment and a minority of the children (9.3%, 8/86) drank untreated water. This distribution was not significantly different for RVA negative children. Comparing RVA-positive and -negative children of different age groups, no significant association was found concerning the drinking water source and the treatment method applied (Table 2).

### Sanitation facilities

Regarding sanitation facilities, 79.5% (272/342) of the children had access to latrines, either private (53.7%, 146/272) or public (46.3%, 126/272). In a significant proportion of these latrines (41.9%, 114/272), there was no running water. So, for a high proportion of children, i.e. 20.5% (70/342), without access to any type of latrines, the most probable option could mean open defecation close to the households. The use of a public latrine with running water determined the highest RVA infection rate in children (35.6%, 21/59). On the other hand, the lowest rate of infection is associated with the absence of latrine (20.0%, 14/70). Nonetheless, no significant difference was observed between the type of sanitation facilities (Table 2) and the rate of RVA infection, on the overall and when considering each age group per se.

### Breastfeeding and nutritional assessment

Breastfeeding practice was reported for 240 (70.2%) children included in the study (of whom 38 were exclusively breastfed and 202 had complementary breastfeeding), while 98 children have already been weaned (28.7%) and 4 have never been breastfed (1.2%). RV infection was significantly more frequent among children who were being breastfed at the time of enrollment (82/240, 34.2%), compared to those that had already been weaned or had never been breastfed (4/102, 3.9%)–p-value <0.001; OR = 12.7; 95% CI [4.518;35.786]. In relation to nutritional assessment, 34.0% (113/332) of the enrolled children were moderate to severe underweighted, 31.0% (103/332) presented moderate to severe wasting and 32.2% (107/332) were moderate to severe stunted. Overall, the percentage of underweighted children (*z* score≤-2) was higher in children with RVA infection as compared to the non-infected (38.8% versus 32.4%, respectively, p = 0.280; CI [0.795; 2.209]), with a higher percentage of underweighted children among RVA infected children between 12–24 months compared to negative cases in the same age group (56.3% and 35.2%, respectively, p = 0.119; CI [0.786;7.116]) (Table 2). However, none of these differences proved to be significant. Similarly, the percentage of wasted children (*z* score≤-2) was higher in children with RVA infection compared to those with a negative result for RVA antigen detection (37.6% and 28.7%, respectively, p = 0.126; CI [0.89;2.513]), more evident for children under twelve months (p = 0.188), although also without significant difference. In contrast, the overall proportion of children stunted (*z* score≤-2) was significantly higher in children not infected with RVA (37.7%) compared to children with RVA positive rapid test (16.5%) (p<0.0001), mainly considering the group age under 12 months (p = 0.006) (Table 2). Stunting continued to be significantly associated with the absence of RVA infection (OR adj = 2.7, 95% CI: 1.390–5.158), considering age, gender and infection by enteric pathogens other than RVA, when a multiple logistic regression model was applied (Hosmer and Lemeshow test, p = 0.681).

### Clinical features associated with RVA infection and hospitalization

With the exception of vomiting, that was significantly associated with RVA infection (p<0.001), the percentage of children exhibiting AGE related symptoms (fever and duration of diarrhea in days) and signs (lethargy, depressed fontanelle, sunken eyes and loss of skin elasticity) was similar in children with or without RVA infection (Table 3). Furthermore, from data available on hospitalization regarding 302 children with AGE, it was observed that 81 (26.8%) were hospitalized, of which 24 (29.6%) were RVA positive cases (Table 3). Hospitalization was non-significantly more common in RVA-positive cases than in RVA-negative cases (32.4% versus 25.0%). Regarding the admission service, there was an excess of RVA positive children at the emergency unit, which proved to be highly significant (p = 0.002) (Table 3).

**Table 3:** Clinical features and hospitalization of children with diarrhea attended at the Bengo General Hospital.

		RV (+)	RV (-)	*p-value*
		n (%)	n (%)	
**Signs and symptoms**	**Vomiting** (N = 342)	N = 86	N = 256	
	**Yes** (N = 175)	62 (72.1)	113 (44.1)	<0.001*
	**No** (N = 167)	24 (27.9)	143 (55.9)	
	**Fever** (N = 342)	N = 86	N = 256	
	**Yes** (N = 255)	69 (80.2)	186 (72.7)	0.163
	**No** (N = 87)	17 (19.8)	70 (27.3)	
	**Duration of diarrhea in days** (N = 333)	N = 85	N = 248	
	**1–3 days** (N = 250)	68 (80.0)	182 (73.4)	0.432
	**4–5 days** (N = 53)	10 (11.8)	43 (17.3)	
	**≥ 6 days** (N = 30)	7 (8.2)	23 (9.3)	
	**Lethargy** (N = 325)	N = 86	N = 239	
	**Yes** (N = 93)	29 (33.7)	64 (26.8)	0.222
	**No** (N = 232)	57 (66.3)	175 (73.2)	
	**Depressed fontanelle** (N = 320)	N = 86	N = 234	
	**Yes** (N = 48)	14 (16.3)	34 (14.5)	0.698
	**No** (N = 272)	72 (83.7)	200 (85.5)	
	**Sunken eyes** (N = 329)	N = 85	N = 244	
	**Yes** (N = 76)	22 (25.9)	54 (22.1)	0.480
	**No** (N = 253)	63 (74.1)	190 (77.9)	
	**Skin (lost of elasticity)** (N = 318)	N = 86	N = 232	
	**Yes** (N = 13)	5 (5.8)	8 (3.4)	0.349
	**No** (N = 305)	81 (94.2)	224 (96.6)	
**Origin and referral of the patient**	**Admission service** (N = 342)	N = 86	N = 256	
	**Outpatient department** (N = 164)	29 (33.7)	135 (52.7)	0.002**
	**Emergency unit** (N = 178)	57 (66.3)	121 (47.3)	
	**Hospitalization** (N = 302)	N = 74	N = 228	
	**Yes** (N = 81)	24 (32.4)	57 (25.0)	0.210
	**No** (N = 221)	50 (67.6)	171 (75.0)	

*OR = 3.3; 95%CI] 1.921–5.564[

**OR = 0.46; 95%CI] 0.274–0.759[

### Seasonality of RVA infection

**Fig 1:**
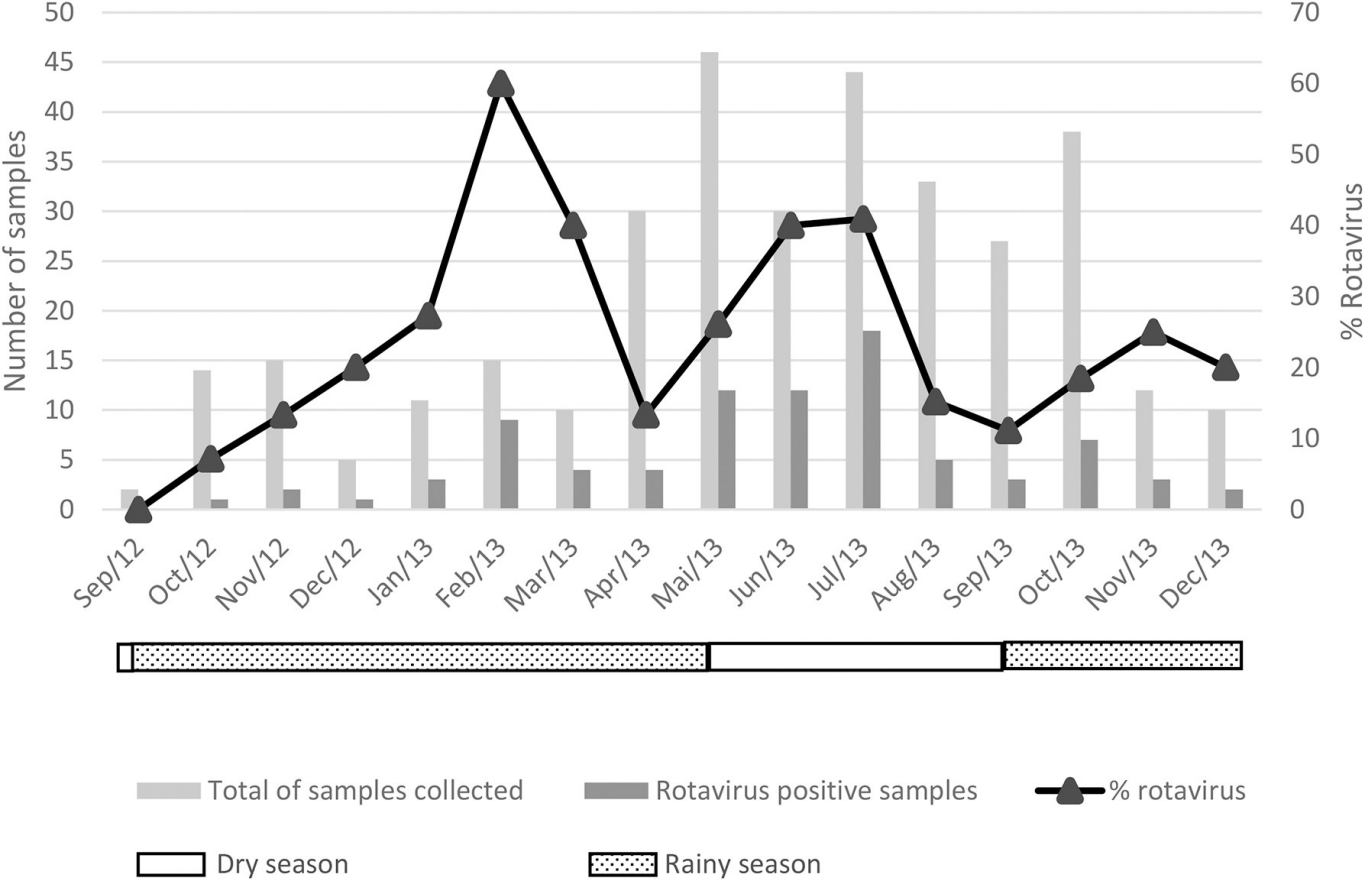
Seasonality of rotavirus a antigen detection in stool samples of children with diarrhea attended at the Bengo General Hospital.

During the study period, there was a substantial increase in diarrhea cases attended at the BGH during the dry season, from May to October, with a peak in May 2013 (46 cases) (Fig 1). RVA infection was detected throughout the study, with higher rates of RVA infection detected in February (60.0%), March (40.0%), June (40.0%) and July (40.9%) 2013 (Fig 1).

### RVA genotypes: High prevalence of G1P[8] and G1P[6]

**Fig 2:**
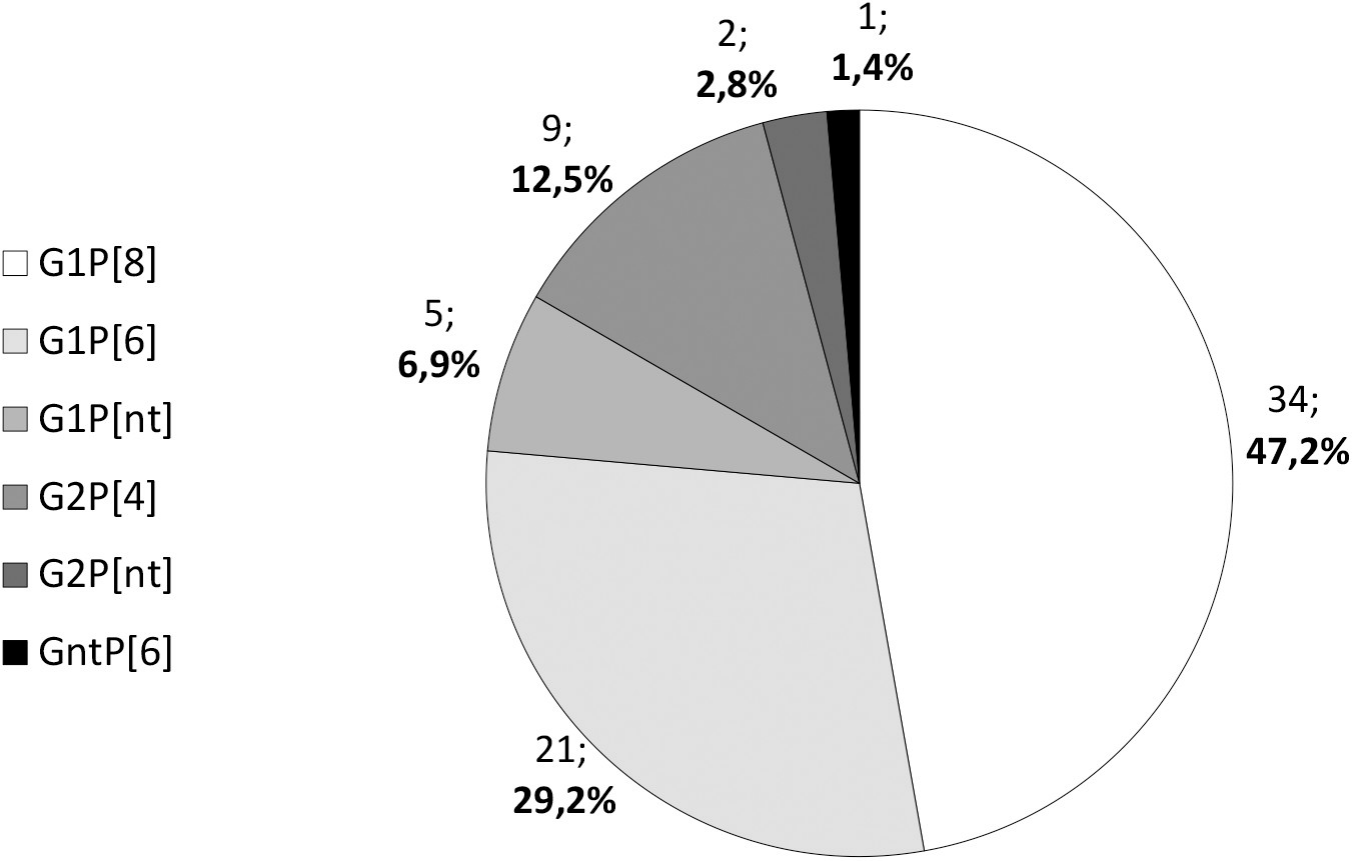
Distribution of G and P genotypes of rotavirus a strains in children with diarrhea attended at the Bengo General Hospital.

From the 86 RVA positive samples detected by rapid immunochromatographic assay, 76 (88.4%) were analyzed by molecular biology methods (the remaining 10 samples had insufficient biological material to proceed with the subsequent analysis). These RVA positive specimens (n = 76) were then further characterized for the VP6, VP4 and VP7 genes by PCR protocols using specific primers [31–34]. Of these, four samples proved to be negative by PCR. For the other 72 samples, the G and P genotypes were determined and results are presented in Fig 2.

The most prevalent genotype combination was G1P[8] (47.2%, 34/72), followed by the unusual G1P[6] (29.2%, 21/72) and G2P[4] (12.5%, 9/72). Only two G-types were found, G1 (83.3%, 60/72) and G2 (15.3%, 11/72), and for one sample it was not possible to determine the G-type. Among the P-genotypes, P[8] was the most prevalent (47.2%, 34/72), followed by P[6] (30.6%, 22/72) and P[4] (12.5%, 9/72). The determination of the P-type was not possible for 7 samples (9.7%, 7/72). When VP6 subgrouping was considered, it was observed that 84.7% (61/72) of the specimens belong to SGII and only 15.3% (11/72) were assigned to SGI. The more frequently detected genotypes G1P[8] and G1P[6] were all assigned to SGII, while G2P[4] to SGI.

### Phylogenetic analysis of VP4 and VP7 genes

**Fig 3:**
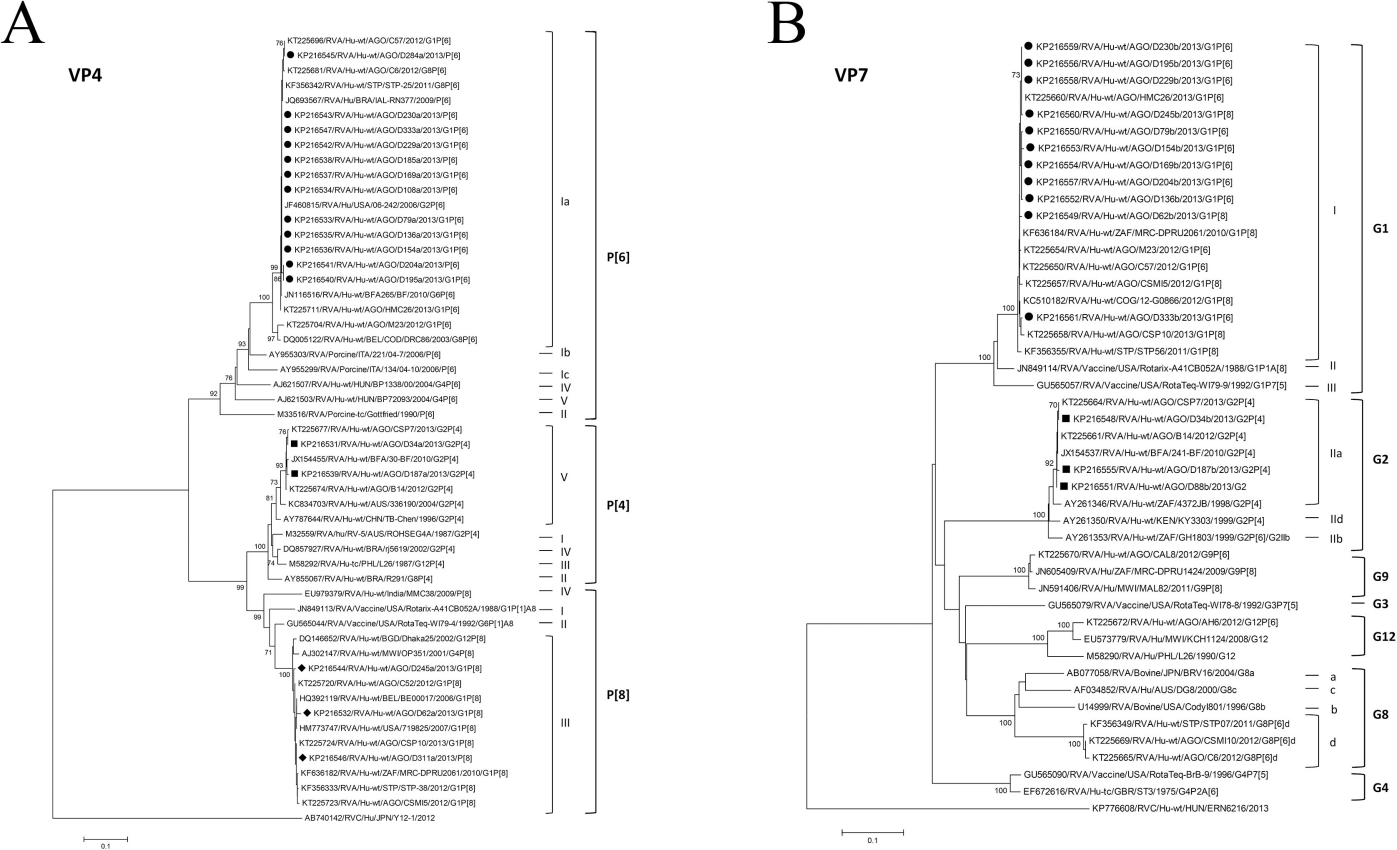
**Phylogenetic analysis of the VP4 (A) and VP7 (B) genes from RVA identified strains.** (A) Genotypes P[6] (closed circles), P[4] (closed squares), and P[8] (closed diamonds) (B) G1 (closed circles) and G2 (closed squares) detected in Bengo, Northwestern Angola. The analysis involved, respectively for VP4 and VP7, 53 and 46 nucleotide sequences and a total of 468 and 627 positions in the final datasets. Phylogenetic analysis was performed using a distance-based neighbour-joining method, based on the Kimura 2-parameter model, with 1,000 bootstrap resamplings (percent bootstrap values of more than 70 are indicated at each node). The tree is drawn to scale, with branch lengths proportional to the evolutionary distances.

Randomly selected PCR amplicons from each identified genotype and from non-typable strains were sent for sequencing. Phylogenetic trees were built with partial reference sequences of VP4 and VP7 genes and are presented in Fig 3A and 3B. The RVA sequences obtained in this study clustered together in well-defined monophyletic groups with reference sequences, with significant bootstrap values (≥92%), defining very robust VP4 and VP7 genotypes.

The Angolan VP4 sequences (n = 17) were assigned to P[4] genotype lineage V (n = 2), P[6] genotype lineage Ia (n = 12) and P[8] genotype lineage III (n = 3) (Fig 3A). The P[4] strains tightly clustered with sequences of African origin, namely from Angola (KT225674, KT225677) and Burkina Faso (JX154455). The P[6] sequences clustered with sequences from Angola (KT225696, KT225681, KT225711) and other sub-Saharan African countries, such as São Tomé and Príncipe (STP) (KF356342) and Burkina Faso (JN116516), as well as with other reference sequences from Brazil (JQ693567) and the USA (JF460815). P[8] sequences, besides with Angolan sequences (KT225720, KT225724, KT225723), and from other African countries, such as STP (KF356333), Malawi (AJ302147) and South Africa (KF636182), clustered with internationally well characterized strains from USA (HM773747), Belgium (HQ392119) and Bangladesh (DQ146652). The P[8] sequences from Bengo clustered all together in the same lineage (3), different from the lineages of the two vaccines Rotarix (lineage 1) and Rotateq (lineage 2).

The Angolan VP7 sequences (n = 14) were assigned to only two genotypes, G1 (lineage I) (n = 11) and G2 (lineage IIa) (n = 3) (Fig 3B). The G1 (lineage I) monophyletic group included the Bengo sequences under study and other sequences from Angola (KT225660, KT225654, KT225650, KT225657, KT225658) and from other African countries, like STP (KF356355), the Democratic Republic of the Congo (KC510182) and South Africa (KF636184). This lineage (I) does not include the two vaccine strains Rotarix (assigned to lineage II) and Rotateq (assigned to lineage III). The three sequences classified as G2 were clustered in lineage IIa with other sequences from Angola (KT225664, KT225661) and Burkina Faso (JX154537).

## Discussion

Although licensed since 2006, rotavirus group A (RVA) vaccines started to be introduced with GAVI support in African countries only since the end of 2011, Sudan being the first designated country. Presently, as many sub-Saharan African countries are still introducing the RVA vaccine in their Expanded Programme on Immunization (EPI), epidemiological studies on RVA burden and disease are still highly important.

In Angola, a leading country in diarrheal diseases, the first studies on this issue were initiated in 2012 in four provinces [22] but neither contemplating Bengo province, nor a year-round sampling. To overcome this, between September 2012 and December 2013, a cross-sectional study was conducted at the Bengo General Hospital (BGH) in Caxito, Bengo province, Angola. RVA detection rate (25.1%) was considered within the range of other African countries [16] although lower than the total detection rate reported for other provinces of the country (35%) [22]. The AdV detection rate was much lower (3.8%), but similar to the rates registered in other sub-Saharan African countries such as Tanzania [38] and the Republic of the Congo [39] before RVA vaccine introduction.

In addition to RVA infection rate (and genotype distribution), we have also investigated several socio-demographic characteristics of the target population trying to get a better insight of the general epidemiology of this virus within this population in Angola.

As shown in most of the African studies including neighboring countries such as Botswana [40] or the Republic of the Congo [39] or considering RVA epidemiology in other provinces of Angola [22], the group of children more significantly affected by RVA AGE was below 12 months of age. This shows a shift of RVA infection towards earlier age in most African settings studied so far, the age factor being considered determinant for vaccination schedule set up, further showing the importance of neonatal vaccine administration in African countries in order to prevent RVA disease [41].

Considering earlier reports which associated maternal literacy and an increased level of education with protection against child mortality and diarrheal diseases [42, 43], we looked into these socio-demographic characteristics, but similarly to Mozambique [44], no correlation was found. Although some studies [45] point towards a correlation between the level of maternal education and some markers of child health, a causal relationship is not unequivocally established.

The high burden of diarrheal diseases, especially affecting developing countries, was previously associated with limited access to drinking water and poor sanitation conditions, factors that facilitate transmission of enteric pathogens [46] thus of RVA. Considering the Global Enteric Multi-Center Study (GEMS) from 2012, in sub-Saharan Africa, 83% of urban and 49% of rural populations have access to improved water sources, while the numbers are much lower when access to improved sanitation facilities is considered (43% of urban and 23% of rural populations) [46]. Improving these conditions became a strong priority and one of the Millennium Development Goals of the WHO. In this study, 28.7% (98/342) of the inquired mothers/caretakers declared to use water from the local Central Water Treatment Plant (CWTP) exactly as supplied, while 59.9% (205/342) applied a domestic treatment, including the use of bleach or alum stone, boiling and/or filtration, to the water provided for the household, irrespective of the source (the CWTP or other sources. i.e., directly from rivers, irrigation channel or boreholes). However, the procedure of home-treatment of drinking water is very difficult to verify (almost impossible to check for the boiling time, if using the correct bleach concentration or type of filters, how to combine with alum stone, etc.) and presumably not always correctly implemented. That is one of the reasons why the results obtained should be taken very cautiously. It is noteworthy that 11.4% (39/342) of the children drank untreated water. As stated before, none of the categories considered seemed to be significantly associated with RVA infection in this study. Comparing proportions of RVA-positive and -negative children of different age groups, no significant association emerged concerning the drinking water source and also the treatment method applied. The lowest RVA detection rate of 20.5% (8/39) was obtained in the group using water originating from alternative sources to the CWTP, with no treatment, and the highest RVA detection rate of 27.6% (27/98) in the group using water from the CWTP with no additional home treatment. Although at first this could mean that children drinking water from CWTP were the most exposed to RVA infection, our statistical analysis does not seem to firmly support that statement.

Despite the absence of any significant differences between the rates of RVA infection by the type of sanitation facilities reported (Table 2), on the overall and when considering each age group, it was interesting to observe that RVA detection rate is the lowest in the absence of latrines (20%, 14/70) and the highest with the use of public latrines (27.8%, 35/126), irrespective of the presence of running water. This could be explained by the fact that latrine sharing creates unsanitary conditions (and keeping the community toilet clean could be difficult under the circumstances), thus creating conditions for a more widespread fecal-oral transmission of enteric pathogens and in this case RVA. To overcome this, proper cleaning and disinfection, implementation of hygienic measures as well as healthy sanitation behavior should be promoted and implemented at household level and particularly at improved public sanitation facilities (use of latrines with running water).

Although no significant association of RVA infection and the drinking water source, water treatment or furthermore hygiene practices related to sanitation facilities of the family inquired was found, we would like to highlight the importance of implementing appropriate and regular “home” treatment of the drinking water, adequate storage and pipeline distribution for the water of the CWTP. We cannot disregard the putative impact of the current poor sanitation conditions on the transmission of other enteric pathogens (not addressed in this study).

The interference of maternal antibodies in breast milk, one of the putative causes that has been put forward to explain RVA vaccine lower efficacy in developing countries, was studied in sub-Saharan settings before [44] and after the vaccine introduction [47]. Recent post-vaccination studies in Zambia and South Africa suggested that lower immunogenicity of Rotarix vaccine could be explained partially by exposure to high antibody titres in breast milk and early exposure to wild-type rotavirus infections [47, 48]. Some pre-vaccination studies from developed [49] as well developing countries [50] indicated that breastfeeding protects infants against AGE caused by RVA. However, our results confirmed the hypothesis presented by a recent study from Mozambique [44] in which no significant protective effect against RVA infection could be associated with breastfeeding.

Rotavirus infection and malnutrition are common in children in the developing countries, thus we investigated the nutritional status of children enrolled. A recent study in Bangladesh showed that a better nutritional status is positively associated with RVA diarrhea in the first three years of life [51]. However, in earlier studies from Africa, wasting was associated with more severe forms of diarrhea and RVA infection [52], showing that the impact of nutritional status on susceptibility to RVA diarrhea is a controversial subject, far from being completely understood. In our study, signs of malnutrition were quite evident, in a high proportion of the children enrolled. Although underweight and wasted children seemed more prone for RVA infection, no significant association was established. On the other hand, stunting (particularly in children below 12 months of age) was significantly more common in children not infected with RVA. Despite the differences observed, we cannot exclude the influence of other factors affecting nutritional status such as maternal stunting and birth weight that were not considered in our study.

In terms of clinical features potentially associated with RVA infection, only vomiting was highly significantly associated with RVA infection. This association was also shown before in other sub-Saharan African countries [52]. As frequent as it may seem in RVA-infected children, clinical management of vomiting requires a special attention, since prolonged vomiting in these small, undernourished, children may rapidly deplete the body of water (dehydration), profoundly impacting the electrolyte equilibrium.

Hospitalization for diarrheal diseases and specifically for RVA infection has been associated with a significant burden to the health systems and households in developing countries [53]. In our study, we observed that the percentage of hospitalization in children with RVA infection (32.4%) was higher than in RVA negative children (25.0%), and that there was a highly significant excess of RVA positive children at the emergency unit, when considering the admission type for the AGE cases at the BGH. Before RVA vaccine introduction, studies to evaluate its economic impact were undertaken. The great benefit of RVA vaccination, e.g. for developing, GAVI-eligible countries, has been shown [54] namely in the reduction of the burden of disease in already fragile healthcare facilities.

Although several studies from sub-Saharan African countries point for a higher prevalence of RVA infection in the dry season [20, 39, 55], no seasonality of RVA infection was evident in our year-round study. Nevertheless, an apparent increase in diarrhea cases admitted at the BGH during the dry season seems plausible (Fig 1), showing the possibility of other enteric agents being involved as etiologic agents.

Currently, a great regional and temporal variety of RVA strains have been reported worldwide [21]. Their relative importance before and after RVA vaccine introduction, especially in developing countries, where concerns aroused about strain replacement by emergence of novel strains which could lower vaccine efficacy, has been acknowledged. Five viral strains were reported as most prevalent globally before RVA vaccine introduction in the national immunization programmes, the most prevalent being G1P[8], identified as dominant also in the scope of this study (47.2%, 34/72). On the overall, this strain is considered as responsible for more than 50% of RVA infections in children and is part of both licensed vaccines (Rotarix and Rotateq), but considering AFROROTANET data a lower detection rate (14–31%) was observed in African countries [16].

Another identified genotype, G1P[6], the second most detected in our study (29.2%, 21/72) has been shown as an emerging strain in recent years. Considering the period 2007–2012, after the RVA vaccines being licensed globally but before introduction in most of African countries, P[6]-type were increasingly detected in Africa, with G1P[6] detection rate established as 6% [21]. After RVA vaccines introduction in African countries, this strain became one of the six most prevalent strains throughout the continent [56], fact that our results seem to corroborate.

Finally, G2P[4], a strain considered predominant as before as after RVA vaccine introduction, registered a detection rate of 12.5% (9/72) in our study. This detection rate is comparable with data reported globally before vaccine introduction (10.6%) [15] but also afterwards (13%, between 2007–2012) [21], as well as with reports from African countries during the period 2007–2013 (10.5%) [56].

The G and P-types identified in this study clustered together respectively with other G1 and G2, and P[4], P[6] and P[8] strains, from sub-Saharan African countries and also international references, sharing a high nucleotide identity with strains detected previously in other provinces of Angola [22]. Albeit the long geographic distances involved, when comparing sampling sites in the two studies, there is a remarkable genetic homogeneity within each genotype, both for VP4 and for VP7 (Fig 3A and 3B). The limited number of genotypes found in this study probably reflects a limited number of recent introductions in the study area of Bengo province in Angola. The identified sequences were assigned to genotypes and furthermore to lineages (e.g. GI-I and P[8]-III) although these lineages are different of the ones included in currently used RVA vaccines (G1-II for Rotarix and G1-III for RotaTeq, P[8]-I for Rotarix and P[8] -II for RotaTeq).

Although P[6] was initially considered of zoonotic origin [18] and assigned to SGI [18, 57], the combination G1P[6] was shown to belong to SGII, as also shown before in the previous Angolan study [22], and in the same subgroup as the well-studied G1P[8].

Finally, in Africa, P[6] RVA infection, described as emerging, has been associated with host genetic factors (e.g. Lewis phenotype), apparently infecting more frequently Lewis-negative children [58]. This Lewis phenotype was also shown to be more common in many African populations than in Caucasian populations of Europe and North America, and thus this fact could explain the increase of this strain detection rate in Africa. Further host genetics studies could elucidate the P[6] association to Lewis-negative phenotype and get an insight on future vaccine efficacy in this population, known the lower efficacy of RVA vaccines in low and middle-income African populations.

## Conclusions

In this hospital-based study, aiming to provide baseline information before vaccine introduction in Angola, the detection rate of RVA infection and genotype distribution in the Bengo province have been investigated. A high detection rate was registered (25.1%) and the most prevalent genotypes were shown to be G1P[8] (47.2%), followed by G1P[6] (29.2%) and the less detected G2P[4] (12.5%). The natural variability of RVA strains in Africa was demonstrated by many studies including the present one, stressing the fact that epidemiological surveillance prior and post RVA vaccine introduction would be crucial to detect potential emergence of new genotypes. Apart from age and age distribution, there was no statistically significant association between RVA infection and any other socio-demographic variable analyzed. Regarding clinical features associated with RVA infection, only vomiting proved to be significantly associated with RVA infection. Although there was an increase in diarrhea cases registered at the BGH during the dry season, no seasonality of RVA infection was proved during the study period.

### Limitations

- Reduced amount of collected sample thus not allowing to genotype all RVA positives detected by immunochromatographic rapid test.
- According to the inclusion criteria set up, children presenting with vomiting only were not enrolled in this study, which may impact on disease burden, underestimating the RVA rate of infection.
